# Emergency Department Use Prior to Cancer Diagnosis and Mortality

**DOI:** 10.1001/jamanetworkopen.2025.22585

**Published:** 2025-07-22

**Authors:** Keerat Grewal, Andrew Calzavara, Shelley L. McLeod, Antoine Eskander, David W. Savage, Cameron Thompson, Bjug Borgundvaag, Howard Ovens, Sheldon Cheskes, Kerstin de Wit, Jonathan C. Irish, Monika K. Krzyzanowska, Rachel Walsh, Venkatesh Thiruganasambandamoorthy, Rinku Sutradhar

**Affiliations:** 1 Schwartz/Reisman Emergency Medicine Institute, Sinai Health, Toronto, Ontario, Canada; 2 Division of Emergency Medicine, Department of Medicine, Temerty Faculty of Medicine, University of Toronto, Toronto, Ontario, Canada; 3 ICES, Toronto, Ontario, Canada; 4 Dalla Lana School of Public Health, University of Toronto, Toronto, Ontario, Canada; 5 Division of Emergency Medicine, Department of Family and Community Medicine, Temerty Faculty of Medicine, University of Toronto, Toronto, Ontario, Canada; 6 Sunnybrook Health Sciences Centre and Odette Cancer Centre, Toronto, Ontario, Canada; 7 Department of Otolaryngology–Head & Neck Surgery, University of Toronto, Toronto, Ontario, Canada; 8 Department of Emergency Medicine, Thunder Bay Regional Health Sciences Centre, Thunder Bay, Ontario, Canada; 9 Division of Clinical Sciences, Northern Ontario School of Medicine (NOSM), Thunder Bay, Ontario, Canada; 10 Sunnybrook Centre for Prehospital Medicine, Sunnybrook Health Sciences Centre, Toronto, Ontario, Canada; 11 Department of Emergency Medicine, Queens University, Kingston, Ontario, Canada; 12 Department of Emergency Medicine, Department of Medicine, McMaster University, Hamilton, Ontario, Canada; 13 Department of Otolaryngology–Head and Neck Surgery, University Health Network, Toronto, Ontario, Canada; 14 Cancer Care Ontario, Toronto, Ontario, Canada; 15 Division of Medical Oncology, Department of Medicine, Temerty Faculty of Medicine, University of Toronto, Toronto, Ontario, Canada; 16 Department of Emergency Medicine, The Ottawa Hospital, Ottawa, Ontario, Canada; 17 Department of Epidemiology and Community Medicine, University of Ottawa, Ottawa, Ontario, Canada

## Abstract

**Importance:**

The emergency department (ED) is a common yet understudied route to cancer diagnosis. It has been reported that over 1 in 3 patients in Ontario, Canada, used the ED prior to cancer diagnosis.

**Objective:**

To examine the association between ED use in the 90 days prior to cancer diagnosis and subsequent mortality.

**Design, Setting, and Participants:**

This matched, retrospective, population-based cohort study used administrative health data from Ontario, Canada. Adults (aged ≥18 years) diagnosed with cancer between January 1, 2014, and December 31, 2021, were included. Patients were followed from index diagnosis until death, 7 years, or end of the study (March 31, 2024). To create the cohort, patients with and without ED use prior to diagnosis were matched 1:1 on sex and year of diagnosis, and then were propensity score matched.

**Exposure:**

Any ED visit in the 90 days prior to diagnosis.

**Main Outcomes and Measures:**

The primary outcome was all-cause mortality after cancer diagnosis. A Cox proportional hazards regression model was used to estimate mortality risk. Interaction with time using restricted cubic splines was included to model the time-varying relationship between ED use and mortality. Results were stratified by hospitalization on the ED visit vs discharge from the ED.

**Results:**

A total of 205 060 (89.3%) patients with ED use prior to cancer diagnosis were matched to patients without ED use prior to diagnosis. Of the 410 120 total patients included, the mean (SD) age was 67.4 (15.0) years, and 106 681 (52.0%) per group were male. Overall mortality was 49.7%: 61.7% in patients with an ED visit vs 37.8% in patients without an ED visit. Patients with ED use prior to diagnosis had statistically significantly higher risk of mortality compared with those without ED use, which decreased with time but persisted through the 7-year follow-up (hazard ratio [HR] at 30 days: 4.49 [95% CI, 4.40-4.58]; HR at 1 year: 1.85 [95% CI, 1.82-1.88]; HR at 3 years: 1.48 [95% CI, 1.46-1.50]; HR at 7 years: 1.05 [95% CI, 1.01-1.09]). In stratified analysis, the increased hazard of death among patients with ED use was even higher among those admitted to the hospital vs the overall model, which persisted over the follow-up time (HR at 30 days: 5.83 [95% CI, 5.69-5.99]; HR at 1 year: 2.23 [95% CI, 2.19-2.27]; HR at 3 years: 1.74 [95% CI, 1.70-1.77]; HR at 7 years: 1.30 [95% CI, 1.23-1.37]). Patients discharged from the ED also had a greater hazard of mortality vs those without ED use, which persisted to 3 years of follow-up (HR at 30 days: 2.68 [95% CI, 2.59-2.77]; HR at 1 year: 1.81 [95% CI, 1.76-1.86]; HR at 3 years: 1.38 [95% CI, 1.34-1.41]; HR at 7 years: 1.03 [95% CI, 0.98-1.10]).

**Conclusions and Relevance:**

In this cohort study of patients diagnosed with cancer, those with ED use prior to cancer diagnosis had a higher hazard of mortality. This finding highlights the need for (1) established systems of care to ensure timely cancer workup for patients in the ED with suspected cancer and (2) health care system improvements to enhance early cancer detection and management to reduce reliance on emergency care for initial cancer presentations.

## Key Points

**Question:**

What is the risk of mortality among patients with and without emergency department (ED) use in the 90 days prior to cancer diagnosis?

**Findings:**

In this cohort study of 410 120 patients with a cancer diagnosis, patients with ED use prior to diagnosis had statistically significantly higher risk of mortality compared with matched patients without ED use, which decreased with time but persisted throughout follow-up.

**Meaning:**

Findings of this study emphasize the need for established systems to ensure timely cancer workup for patients in the ED with suspected cancer and the need for health care system improvements to enhance early cancer detection and management.

## Teaser

This cohort study compares risk of mortality between patients diagnosed with cancer who visited the emergency department (ED) in the 90 days prior to diagnosis with those who had no ED visit.

## Introduction

Patients with symptoms suggestive of cancer are commonly seen in the emergency department (ED) prior to diagnosis. Cancer is often initially suspected through the ED based on suspicious imaging, blood work, physical examination, and/or symptoms, requiring further workup to confirm a cancer diagnosis.^1,2,3^ This route to diagnosis has been reported globally.^2,3,4,5^ A recent study reported that over one-third of patients diagnosed with cancer in Ontario, Canada, used the ED in the 90 days before their cancer diagnosis.^5^

Previous studies have found that patients who are diagnosed with cancer through the ED have worse outcomes.^6,7,8^ These patients present with advanced stage at diagnosis and have worse overall survival compared with patients diagnosed through other routes. The excess mortality observed in patients with ED involvement in a cancer diagnosis has been reported to be independent of cancer stage^8^ and observed across different cancer types.^6,7^

Although patients with suspected cancer are seen in the ED frequently, few studies have examined long-term outcomes among patients diagnosed with cancer after an ED visit. Studies that have examined outcomes among patients with ED involvement in a cancer diagnosis have largely been focused on specific types of cancer,^8,9,10^ included patients who required emergency admission to hospital, consisted of small cohorts,^9,10^ or assessed short-term mortality.^8^ The objective of this study was to examine the association between ED use in the 90 days prior to cancer diagnosis and mortality in a large population-based cohort of patients diagnosed with any cancer in Ontario, Canada.

## Methods

### Study Design and Setting

We conducted a retrospective matched cohort study using population-based administrative health data from Ontario, Canada, from 2014 to 2024. Data were obtained from provincial databases housed at ICES. Ontario has universal health care coverage for medically necessary care; therefore, these databases contain most health care use. ICES is a prescribed entity under Ontario’s Personal Health Information Protection Act (PHIPA). Projects that use data collected by ICES under section 45 of PHIPA and that use no other data are exempt from Research Ethics Board review and the informed consent requirement. The use of the data in this study is authorized under section 45 and was approved by the ICES Privacy and Legal Office. We followed the Strengthening the Reporting of Observational Studies in Epidemiology (STROBE) reporting guideline.^11^

### Data Sources

Patients with a confirmed diagnosis of cancer were identified from the Ontario Cancer Registry (OCR). The OCR contains information on all diagnosed cases of cancer in Ontario (except basal and squamous cell skin cancers). The Canadian Institute for Health Information (CIHI) National Ambulatory Care Reporting System was used to identify information regarding ED visits in Ontario. The CIHI Discharge Abstract Database was used to identify hospitalizations. The Ontario Health Insurance Plan database contains physician billings for medically necessary care. The Immigration, Refugees and Citizenship Canada Permanent Resident Database was used to identify immigration status. The Registered Persons Database contains demographics and vital statistics for all patients, including mortality for Ontario residents and out-of-hospital deaths. These datasets were linked using unique encoded identifiers and analyzed at ICES. The eMethods in Supplement 1 provides additional information about datasets.

### Study Participants

Adult patients 18 years or older with a confirmed cancer diagnosis between January 1, 2014, and December 31, 2021, were identified from the OCR. The diagnosis date in the OCR is based on the pathology date of diagnosis. The first primary cancer diagnosis for each patient during this period was used. Patients with a death date up to 90 days prior to the cancer diagnosis date (negative death date) were included to allow for postmortem diagnosis or small discrepancies in recorded dates and were assigned a survival time of 0.5 days. Patients without a valid Ontario Health Insurance Plan number or with a recorded date of death occurring more than 90 days prior to the cancer diagnosis date were excluded.

### Exposure and Outcome Variables

The exposure variable of interest was any ED visit in the 90 days prior to cancer diagnosis, which was treated as a dichotomous variable. We identified the first cancer diagnosis in the OCR between 2014 and 2021 and then looked back 90 days from the OCR diagnosis date to identify patients with any ED visit prior to the cancer diagnosis date. Based on previous work examining ED use prior to cancer diagnosis, we used the 90-day window to capture ED use that was more likely associated with a potential cancer diagnosis.^5^ For patients with multiple ED visits, the visit closest to the cancer diagnosis date was used. The outcome of interest was time to all-cause mortality, starting from the date of cancer diagnosis.

### Covariates

Covariates of interest were potential factors and confounders associated with ED use prior to cancer diagnosis and mortality. These covariates included demographics (age, sex, immigration in the past 5 years, rural residence, and Ontario Health region), marginalization (area-level material resources and household and dwellings stability), previous health care use (hospitalizations and ED visits prior to cancer diagnosis and continuity of medical care), comorbidities (coronary artery disease, congestive heart failure, chronic obstructive pulmonary disease, diabetes, dementia, hypertension, and stroke), cancer type, and diagnosis year. All covariates were measured at the time of diagnosis. We used 2 components of the Ontario Marginalization Index as markers of socioeconomic status or marginalization: the material resources and household and dwellings stability.^12^ These components were reported as quintiles, with the higher quintiles representing more marginalized individuals.^12^

We defined continuity of medical care based on the Usual Provider of Care (UPC) Index.^13,14^ We examined ambulatory visits in the 6 to 30 months prior to diagnosis and classified patients as follows: (1) with a UPC (>50% of visits) who was a general practitioner or primary care physician, (2) with a UPC who was a specialist, (3) without a UPC but with a family physician visit, (4) without a UPC and without a family physician visit, (5) with 1 to 2 ambulatory visits, and (6) without ambulatory visits. For previous health care use and continuity of medical care, we examined health care use in the 6 to 30 months prior to cancer diagnosis to allow for a 6-month washout period to ensure that cancer-related health care use was not captured. The eMethods in Supplement 1 provides further covariate definitions.

### Statistical Analysis

Before and after matching, distributions of baseline characteristics were compared between patients with and without an ED visit in the 90 days before a cancer diagnosis using standardized differences. Standardized differences less than 0.10 indicated balance between groups.^15^ To create the 1:1 matched cohort, patients with vs without an ED visit prior to cancer diagnosis were hard matched on sex and year of diagnosis. Propensity score matching was also used to match patients with vs without an ED visit on covariates that were not balanced at baseline, based on standardized differences greater than 0.10 prior to matching. The propensity score was estimated using logistic regression, where the exposure status (ED visit or no ED visit prior to cancer diagnosis) was regressed on the covariates that exhibited lack of balance. Covariates included in the propensity score were age (as a cubic function), sex, cancer type, year of diagnosis, rurality, marginalization index, previous hospitalizations, previous ED visits, and comorbidities. The remaining covariates (immigration in the past 5 years, Ontario Health region, and UPC type) were well balanced prior to matching and not included in the propensity score. Patients in each exposure group were matched on the logit of the propensity score using a 1:1 without-replacement approach and a caliper width of 0.2 of the SD of the logit of the propensity score.

The cumulative incidence function was used to estimate the risk of all-cause mortality over time. With the date of cancer diagnosis serving as the index date, we used a Cox proportional hazards regression model to compare the relative hazard of death between patients with and without an ED visit prior to cancer diagnosis. A robust sandwich variance estimator was used to account for the matched nature of the cohort. Patients were followed from index until death or right censoring, which occurred at the 7-year mark (2555 days) or the end of the study period (March 31, 2024). Due to violation of the proportional hazards assumption for the primary exposure, an interaction with time modeled using restricted cubic splines was included in the unadjusted and adjusted regressions to examine the time-varying relationship between ED use and mortality. The spline was specified with 4 knots placed to create 5 time intervals that each contained an equal number of deaths. Unadjusted and adjusted hazard ratios (HRs) and 95% CIs were calculated. The adjusted model included covariates that remained unbalanced after matching (only the UPC variable).

We performed 2 sensitivity analyses. First, we repeated the primary analysis after excluding matched pairs comprising 1 or both patients diagnosed on or after the date of death. Second, we stratified the exposed patients (with ED visit prior to diagnosis) who were hospitalized at the ED visit vs discharged from the ED. The matches (with corresponding controls) were retained in the stratified analysis. Analyses were conducted from April 2024 to April 2025 using SAS, version 9.4 (SAS Institute).

## Results

As previously reported,^5^ we identified 651 071 patients diagnosed with cancer in Ontario between 2014 and 2021. Of these patients, 229 683 (35.3%) had an ED visit in the 90 days prior to their cancer diagnosis, with 118 072 (51.4%) admitted to hospital. Table 1 presents the distributions of baseline characteristics of patients, with standardized differences, prior to matching.

**Table 1.** Baseline Characteristics Before Matching of Patients With and Without an ED Visit in the 90 Days Prior to Cancer Diagnosis

Characteristic	Patients, No. (%)			Standardized difference
	Total (n = 651 071)	ED visit in the 90 d prior to cancer diagnosis		
		Without visit (n = 421 388)	With visit (n = 229 683)	
Age group, y				
18-54	153 432 (23.6)	115 963 (27.5)	37 469 (16.3)	0.27
55-64	138 602 (21.3)	95 564 (22.7)	43 038 (18.7)	0.10
65-74	174 941 (26.9)	115 873 (27.5)	59 068 (25.7)	0.04
≥75	184 096 (28.3)	93 988 (22.3)	90 108 (39.2)	0.37
Sex				
Female	352 722 (54.2)	241 897 (57.4)	110 825 (48.3)	0.18
Male	298 349 (45.8)	179 491 (42.6)	118 858 (51.7)	0.18
Rural				
No	585 314 (89.9)	383 355 (91.0)	201 959 (87.9)	0.10
Yes	64 304 (9.9)	37 095 (8.8)	27 209 (11.8)	0.10
Missing data	1453 (0.2)	938 (0.2)	515 (0.2)	0.00
Material resources quintile				
1 (highest resources)	134 622 (20.7)	94 382 (22.4)	40 240 (17.5)	0.12
2	133 195 (20.5)	89 755 (21.3)	43 440 (18.9)	0.06
3	127 761 (19.6)	82 888 (19.7)	44 873 (19.5)	0.00
4	124 532 (19.1)	77 587 (18.4)	46 945 (20.4)	0.05
5 (lowest resources)	125 438 (19.3)	73 612 (17.5)	51 826 (22.6)	0.13
Missing data	5523 (0.8)	3164 (0.8)	2359 (1.0)	0.03
Household and dwellings stability quintile				
1 (highest stability)	111 658 (17.1)	78 143 (18.5)	33 515 (14.6)	0.11
2	119 552 (18.4)	80 448 (19.1)	39 104 (17.0)	0.05
3	125 558 (19.3)	81 029 (19.2)	44 529 (19.4)	0.00
4	128 519 (19.7)	80 338 (19.1)	48 181 (21.0)	0.05
5 (lowest stability)	160 261 (24.6)	98 266 (23.3)	61 995 (27.0)	0.09
Missing data	5523 (0.8)	3164 (0.8)	2359 (1.0)	0.03
Immigration in past 5 y				
No	639 690 (98.3)	412 697 (97.9)	226 993 (98.8)	0.02
Yes	11 381 (1.7)	8691 (2.1)	2690 (1.2)	0.07
Ontario Health region				
West	196 848 (30.2)	125 921 (29.9)	70 927 (30.9)	0.02
Central	145 276 (22.3)	97 978 (23.3)	47 298 (20.6)	0.06
Toronto	119 955 (18.4)	79 394 (18.8)	40 561 (17.7)	0.03
East	142 944 (22.0)	91 773 (21.8)	51 171 (22.3)	0.01
North East	33 665 (5.2)	19 318 (4.6)	14 347 (6.2)	0.07
North West	12 147 (1.9)	6791 (1.6)	5356 (2.3)	0.05
Missing data	236 (0.0)	213 (0.1)	23 (0.0)	0.02
Comorbidities				
CAD	79 737 (12.2)	42 074 (10.0)	37 663 (16.4)	0.19
CHF	51 501 (7.9)	20 482 (4.9)	31 019 (13.5)	0.30
COPD	64 365 (9.9)	29 613 (7.0)	34 752 (15.1)	0.26
Dementia	26 248 (4.0)	10 622 (2.5)	15 626 (6.8)	0.20
Diabetes	154 141 (23.7)	86 994 (20.6)	67 147 (29.2)	0.20
Hypertension	348 870 (53.6)	206 392 (49.0)	142 478 (62.0)	0.27
Stroke	22 410 (3.4)	9484 (2.3)	12 926 (5.6)	0.17
Any ED visit 6-30 mo preindex diagnosis	276 869 (42.5)	159 228 (37.8)	117 641 (51.2)	0.27
Any hospitalization 6-30 mo preindex diagnosis	101 153 (15.5)	53 870 (12.8)	47 283 (20.6)	0.21
UPC type				
Without ambulatory visits	36 645 (5.6)	21 083 (5.0)	15 562 (6.8)	0.08
With 1-2 ambulatory visits	46 032 (7.1)	29 907 (7.1)	16 125 (7.0)	0.00
Without UPC, without GP visits	3474 (0.5)	2120 (0.5)	1354 (0.6)	0.01
Without UPC, with GP visits	227 674 (35.0)	149 592 (35.5)	78 082 (34.0)	0.03
With UPC, specialist	47 428 (7.3)	31 080 (7.4)	16 348 (7.1)	0.01
With UPC, GP	289 818 (44.5)	187 606 (44.5)	102 212 (44.5)	0.00
Cancer type				
Bladder or urinary	33 040 (5.1)	20 535 (4.9)	12 505 (5.4)	0.03
Breast	88 446 (13.6)	76 923 (18.3)	11 523 (5.0)	0.42
Colorectal or small intestinal	68 563 (10.5)	37 474 (8.9)	31 089 (13.5)	0.15
Gastroesophageal	17 991 (2.8)	8440 (2.0)	9551 (4.2)	0.13
Gynecological	84 536 (13.0)	67 089 (15.9)	17 447 (7.6)	0.26
Head or neck	41 652 (6.4)	32 921 (7.8)	8731 (3.8)	0.17
Hematologic or lymphoma	64 850 (10.0)	32 761 (7.8)	32 089 (14.0)	0.20
Liver or gall bladder	15 047 (2.3)	5474 (1.3)	9573 (4.2)	0.18
Male genitourinary	68 657 (10.5)	55 195 (13.1)	13 462 (5.9)	0.25
Neurological	8218 (1.3)	1287 (0.3)	6931 (3.0)	0.21
Other or unknown	55 125 (8.5)	37 566 (8.9)	17 559 (7.6)	0.05
Pancreatic	15 580 (2.4)	4509 (1.1)	11 071 (4.8)	0.22
Renal	17 925 (2.8)	10 800 (2.6)	7125 (3.1)	0.03
Thoracic	71 441 (11.0)	30 414 (7.2)	41 027 (17.9)	0.33
Diagnosis year				
2014	80 240 (12.3)	52 010 (12.3)	28 230 (12.3)	0.00
2015	81 389 (12.5)	52 791 (12.5)	28 598 (12.5)	0.00
2016	82 383 (12.7)	53 927 (12.8)	28 456 (12.4)	0.01
2017	84 503 (13.0)	55 137 (13.1)	29 366 (12.8)	0.01
2018	83 928 (12.9)	54 990 (13.0)	28 938 (12.6)	0.01
2019	83 469 (12.8)	54 260 (12.9)	29 209 (12.7)	0.01
2020	74 135 (11.4)	45 513 (10.8)	28 622 (12.5)	0.05
2021	81 024 (12.4)	52 760 (12.5)	28 264 (12.3)	0.01

Abbreviations: CAD, coronary artery disease; CHF, congestive heart failure; COPD, chronic obstructive pulmonary disease; ED, emergency department; GP, general practitioner; UPC, usual provider of care.

We matched 205 060 patients (89.3%) with ED use prior to cancer diagnosis to those without ED use prior to diagnosis, resulting in a total cohort of 410 120 patients (eFigure 1 in Supplement 1). Both the exposed group and the matched controls included 106 681 (52.0%) males and 98 379 (48.0%) females. The exposed group had a mean (SD) age of 67.6 (15.3) years, and the matched controls had a mean (SD) age of 67.2 (14.7) years. After matching, patients with vs without an ED visit prior to cancer diagnosis were well balanced, except for the UPC variable, which was then included in the adjusted model. Table 2 provides the baseline characteristics of matched patients. The eTable in Supplement 1 shows the characteristics of unmatched patients.

**Table 2.** Baseline Characteristics After Matching of Patients With and Without an ED Visit Prior to Cancer Diagnosis

Characteristic	Patients, No. (%)		Standardized difference
	With ED visit in the 90 d prior to cancer diagnosis (n = 205 060)	Without ED visit in the 90 d prior to cancer diagnosis (n = 205 060)	
Age group, y			
18-54	35 925 (17.5)	35 401 (17.3)	0.01
55-64	40 439 (19.7)	41 667 (20.3)	0.02
65-74	54 614 (26.6)	59 802 (29.2)	0.06
≥75	74 082 (36.1)	68 190 (33.3)	0.06
Sex			
Female	98 379 (48.0)	98 379 (48.0)	0.00
Male	106 681 (52.0)	106 681 (52.0)	0.00
Rural			
No	181 084 (88.3)	182 139 (88.8)	0.02
Yes	23 509 (11.5)	22 473 (11.0)	0.02
Missing data	467 (0.2)	448 (0.2)	0.00
Material resources quintile			
1 (highest resources)	37 002 (18.0)	38 074 (18.6)	0.01
2	39 475 (19.3)	40 249 (19.6)	0.01
3	40 348 (19.7)	40 912 (20.0)	0.01
4	41 637 (20.3)	41 120 (20.1)	0.01
5 (lowest resources)	44 604 (21.8)	42 839 (20.9)	0.02
Missing data	1994 (1.0)	1866 (0.9)	0.01
Household and dwellings stability quintile			
1 (highest stability)	30 888 (15.1)	31 926 (15.6)	0.01
2	35 730 (17.4)	36 545 (17.8)	0.01
3	39 951 (19.5)	40 228 (19.6)	0.00
4	42 578 (20.8)	42 207 (20.6)	0.00
5 (lowest stability)	53 919 (26.3)	52 288 (25.5)	0.02
Missing data	1994 (1.0)	1866 (0.9)	0.01
Immigration in past 5 y			
No	170 614 (83.2)	169 666 (82.7)	0.01
Yes	2055 (1.0)	2533 (1.2)	0.02
Unknown	32 391 (15.8)	32 861 (16.0)	0.01
Ontario Health region			
West	63 304 (30.9)	63 629 (31.0)	0.00
Central	42 817 (20.9)	43 791 (21.4)	0.01
Toronto	35 892 (17.5)	37 059 (18.1)	0.02
East	45 788 (22.3)	45 621 (22.2)	0.00
North East	12 513 (6.1)	11 064 (5.4)	0.03
North West	4724 (2.3)	3800 (1.9)	0.03
Missing data	22 (0.0)	96 (0.0)	0.02
Comorbidities			
CAD	31 289 (15.3)	28 884 (14.1)	0.03
CHF	22 171 (10.8)	17 703 (8.6)	0.07
COPD	27 656 (13.5)	23 620 (11.5)	0.06
Dementia	10 989 (5.4)	8896 (4.3)	0.05
Diabetes	56 971 (27.8)	54 385 (26.5)	0.03
Hypertension	123 121 (60.0)	120 351 (58.7)	0.03
Stroke	9362 (4.6)	7733 (3.8)	0.04
Any ED visit 6-30 mos preindex diagnosis	100 014 (48.8)	95 190 (46.4)	0.05
Any hospitalization 6-30 mos preindex diagnosis	38 225 (18.6)	34 496 (16.8)	0.05
UPC type			
Without ambulatory visits	14 938 (7.3)	8719 (4.3)	0.13
With 1-2 ambulatory visits	15 127 (7.4)	11 987 (5.8)	0.06
Without UPC, without GP visits	1185 (0.6)	1265 (0.6)	0.01
Without UPC, with GP visits	68 513 (33.4)	76 724 (37.4)	0.08
With UPC, specialist	14 844 (7.2)	14 476 (7.1)	0.01
With UPC, GP	90 453 (44.1)	91 889 (44.8)	0.01
Cancer type			
Bladder or urinary	12 170 (5.9)	14 095 (6.9)	0.04
Breast	11 520 (5.6)	10 960 (5.3)	0.01
Colorectal or small intestinal	28 960 (14.1)	33 101 (16.1)	0.06
Gastroesophageal	8470 (4.1)	8397 (4.1)	0.00
Gynecological	17 392 (8.5)	16 911 (8.2)	0.01
Head or neck	8712 (4.2)	8712 (4.2)	0.00
Hematologic or lymphoma	28 774 (14.0)	30 998 (15.1)	0.03
Liver or gall bladder	7336 (3.6)	5474 (2.7)	0.05
Male genitourinary	13 430 (6.5)	12 975 (6.3)	0.01
Neurological	2456 (1.2)	1284 (0.6)	0.06
Other or unknown	17 133 (8.4)	18 728 (9.1)	0.03
Pancreatic	7056 (3.4)	4509 (2.2)	0.08
Renal	6902 (3.4)	8502 (4.1)	0.04
Thoracic	34 749 (16.9)	30 414 (14.8)	0.06
Diagnosis year			
2014	25 091 (12.2)	25 091 (12.2)	0.00
2015	25 242 (12.3)	25 242 (12.3)	0.00
2016	25 664 (12.5)	25 664 (12.5)	0.00
2017	26 161 (12.8)	26 161 (12.8)	0.00
2018	25 969 (12.7)	25 969 (12.7)	0.00
2019	26 345 (12.8)	26 345 (12.8)	0.00
2020	24 774 (12.1)	24 774 (12.1)	0.00
2021	25 814 (12.6)	25 814 (12.6)	0.00

Abbreviations: CAD, coronary artery disease; CHF, congestive heart failure; COPD, chronic obstructive pulmonary disease; ED, emergency department; GP, general practitioner; UPC, usual provider of care.

The overall risk of mortality over the 7-year period was 49.7% (203 957 deaths): 61.7% in patients with an ED visit prior to cancer diagnosis vs 37.8% in patients without an ED visit prior to cancer diagnosis (Figure). Patients with ED use prior to cancer diagnosis had a statistically significantly higher mortality risk compared with those without ED use. This relative difference decreased with time but persisted over the 7 years of study (HR at 30 days: 4.49 [95% CI, 4.40-4.58]; HR at 1 year: 1.85 [95% CI, 1.82-1.88]; HR at 3 years: 1.48 [95% CI, 1.46-1.50]; HR at 7 years: 1.05 [95% CI, 1.01-1.09]). The sensitivity analysis, which excluded 6248 pairs (3.1% of the matched cohort) with a diagnosis on the date of death, showed results similar to those of the main analysis. Table 3 presents the unadjusted and adjusted models and sensitivity analysis.

**Figure.**
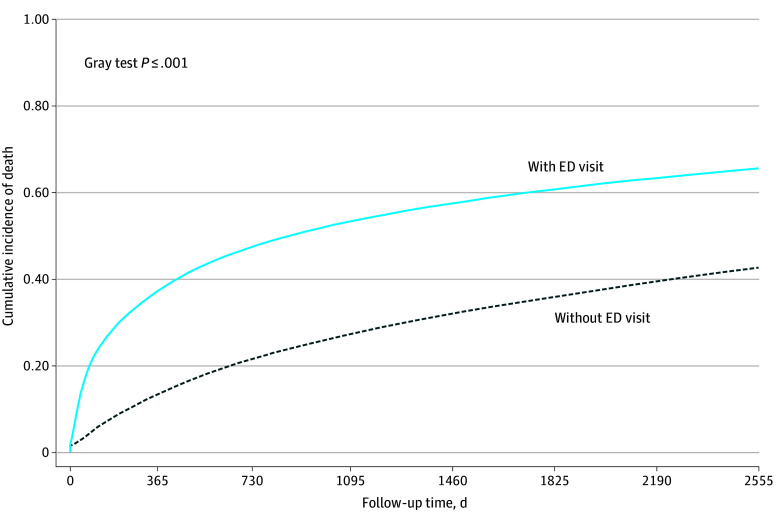
Cumulative Incidence Function for All-Cause Mortality ED indicates emergency department.

**Table 3.** Hazard of Death at Various Time Points for Patients With vs Without an Emergency Department Visit Prior to Cancer Diagnosis

Time point, d	Mortality HR (95% CI)		
	Unadjusted model (n = 410 120)	Adjusted model (n = 410 120)^a^	Sensitivity analysis, excluding patients with negative death days
30	4.48 (4.39-4.57)	4.49 (4.40-4.58)	6.13 (5.99-6.28)
90	3.51 (3.46-3.56)	3.52 (3.47-3.57)	4.25 (4.18-4.32)
180	2.54 (2.49-2.58)	2.55 (2.50-2.59)	2.58 (2.54-2.63)
270	2.06 (2.02-2.10)	2.07 (2.03-2.11)	1.89 (1.85-1.93)
365 (1 y)	1.85 (1.82-1.88)	1.85 (1.82-1.88)	1.67 (1.64-1.70)
545	1.70 (1.68-1.73)	1.71 (1.68-1.74)	1.67 (1.65-1.70)
730 (2 y)	1.61 (1.58-1.64)	1.62 (1.59-1.65)	1.66 (1.63-1.70)
1095 (3 y)	1.48 (1.45-1.50)	1.48 (1.46-1.50)	1.52 (1.50-1.54)
2555 (7 y)	1.05 (1.00-1.09)	1.05 (1.01-1.09)	1.00 (0.96-1.04)

Abbreviation: HR, hazard ratio.

^a^ Adjusted for the usual provider of care type.

In the stratified analysis, both patients hospitalized at the ED visit and those discharged from the ED had a higher risk of death compared with patients who did not use the ED prior to cancer diagnosis. Patients who were admitted to the hospital at the ED visit had higher HRs at all time points compared with the overall model, which also persisted throughout the 7-year follow-up (HR at 30 days: 5.83 [95% CI, 5.69-5.99]; HR at 1 year: 2.23 [95% CI, 2.19-2.27]; HR at 3 years: 1.74 [95% CI, 1.70-1.77]; HR at 7 years: 1.30 [95% CI, 1.23-1.37]). Patients discharged from the ED also had higher risk of mortality vs those without ED use but not as high as in the overall model, and this result persisted to 3 years of follow-up (HR at 30 days: 2.68 [95% CI, 2.59-2.77]; HR at 1 year: 1.81 [95% CI, 1.76-1.86]; HR at 3 years: 1.38 [95% CI, 1.34-1.41]; HR at 7 years: 1.03 [95% CI, 0.98-1.10]) (Table 4). The eFigure 2 in Supplement 1 provides results from stratified analyses.

**Table 4.** Stratified Analysis of Death for Exposed Patients Hospitalized vs Discharged on the Emergency Department Visit Compared With Matched Patients Without an Emergency Department Visit Prior to Cancer Diagnosis

Time point, d	Mortality HR (95% CI)	
	Hospitalized patients (n = 200 516)	Discharged patients (n = 209 154)
30	5.83 (5.69-5.99)	2.68 (2.59-2.77)
90	4.27 (4.19-4.36)	2.48 (2.42-2.54)
180	3.00 (2.91-3.08)	2.21 (2.16-2.25)
270	2.47 (2.41-2.53)	1.98 (1.93-2.04)
365 (1 y)	2.23 (2.19-2.27)	1.81 (1.76-1.86)
545	1.99 (1.95-2.04)	1.62 (1.58-1.66)
730 (2 y)	1.87 (1.83-1.92)	1.51 (1.47-1.55)
1095 (3 y)	1.74 (1.70-1.77)	1.38 (1.34-1.41)
2555 (7 y)	1.30 (1.23-1.37)	1.03 (0.98-1.10)

Abbreviation: HR, hazard ratio.

## Discussion

In this large, population-based study of patients diagnosed with cancer in Ontario, ED use in the 90 days before cancer diagnosis was associated with an increased hazard of mortality compared with matched patients without ED use prior to cancer diagnosis. This risk of mortality persisted in the long-term and was amplified among patients who required hospital admission from the ED visit. To our knowledge, this study is one of the largest examining this issue and reporting on all cancers.

The results of our study are consistent with studies from other countries. In a study from the UK that examined patients with cervical, colorectal, breast, lung, and prostate cancer, excess mortality was observed among patients diagnosed with cancer through the ED. The excess mortality was independent of stage and other case-mix factors.^8^ A study from the US reported an adjusted odds of mortality of 4.12 (95% CI, 3.72-4.56) among patients with ED use in the 6 months prior to cancer diagnosis compared with those without an ED visit.^2^ Two recent reviews reported worse overall survival among patients with ED involvement in diagnosis across several types of cancers and countries.^6,7^ Advanced cancer stage at diagnosis among patients with ED use may be associated with the increased mortality observed in these patients.^9,10,16,17^ Advanced disease may produce signs or symptoms that necessitate an ED visit. With the databases used in our study, we were unable to match patients based on stage at diagnosis due to a large proportion of missing data. However, as described, some studies have suggested that the increased mortality observed among patients diagnosed with cancer through the ED is independent of stage.^8^ In this study, both patients who were admitted to the hospital from the ED and those who were well enough to be discharged from the ED had higher risk of mortality, suggesting that worse mortality is not only restricted to the patients who are acutely unwell from cancer presentation requiring hospitalization.

It is imperative to understand ED use during the diagnostic phase of the cancer trajectory, particularly because of the worse outcomes among patients with ED involvement in cancer diagnosis seen in our study and other studies. While some ED use during the diagnostic phase of cancer may be unavoidable, there are other visits that may be preventable and associated with poor access to care in other ambulatory settings. Delamare Fauvel et al^7^ described 3 ways that the ED may play a role in cancer diagnosis: (1) incidental findings of suspected cancer for unrelated complaints, (2) presentation of cancer with acute signs and symptoms requiring emergency care, and (3) acceleration of a cancer diagnosis and further care. In Europe, diagnosis of cancer through the ED is an indicator of diagnostic quality.^18^ Therefore, improved access to ambulatory health care, such as primary care, specialist care, and imaging along with ensuring uptake of cancer screening programs is needed to reduce reliance on EDs during diagnosis. The ED is often not the ideal environment to facilitate further cancer workup, given the lack of care continuity, variable management of patients, and the chaotic environment of this setting.^19,20^ Therefore, solutions to reduce the need for ED use during diagnosis are needed.

Given the large proportion of patients who use the ED prior to cancer diagnosis and their worse outcomes, ensuring timely access to follow-up care from the ED to confirm a cancer diagnosis is needed. A recent study reported considerable variation in patient management after an ED visit where cancer is suspected, which is often dependent on local resources available at the hospital or in the community the patient is seen and the type of cancer the patient may have.^20^ Qualitative work shows that emergency physicians often find it challenging to determine the appropriate next steps for ensuring timely workup for patients with suspected cancer (personal communication with C. Moore, MSc, email, November 2024).^21^ Therefore, system-level solutions are needed to provide equitable and timely access to further cancer workup for patients seen in the ED with suspected cancer diagnosis. These solutions may include rapid diagnostic clinics, which exist for some types of cancer but are not widely available; central intake for referrals; and navigation from the ED. The diagnostic phase of the cancer trajectory has been described as anxiety provoking with many unknowns for patients, including when they will be seen, what treatment will entail, and prognosis.^22^ Ensuring patients have access to timely and reliable workup from the ED along with clear information about next steps may help reduce some of these unknowns from the ED during this period.

### Limitations

There are several limitations with this study. We were unable to identify cancer-related ED visits and examined any ED use in the 90-day prior to cancer diagnosis. Therefore, some ED visits included may not have been related to cancer diagnosis. Since this was an observational study, we cannot establish causality between ED visits before cancer diagnosis and mortality, and confounding factors may still have affected the observed association despite matching and adjustments. We were unable to account for cancer stage at diagnosis, which may be associated with mortality. However, we were able to account for cancer type, comorbidities, health care utilization, and socioeconomic status. We did not examine race and ethnicity, which may also affect outcomes. The study focused on ED use prior to cancer diagnosis and subsequent mortality but did not examine postdiagnosis care such as treatment that may change survival. We did not include all patients in the matched cohort; however, we achieved an excellent match rate of 89.3%. Characteristics of unmatched patients indicate that the results are likely biased toward the null; however, we still observed a large disparity in mortality. We examined all cancers, and there may be cancer-specific differences in outcomes; thus, future work examining the differences among each type of cancer is needed. We examined health care use by patients in Ontario, Canada, which may limit the generalizability of findings to other jurisdictions.

## Conclusions

Patients presenting to the ED prior to cancer diagnosis face substantially higher mortality risk. This result emphasizes the need for (1) established systems of care to ensure timely cancer workup for patients with suspected cancer in the ED and (2) health care system improvements to enhance early cancer detection and management, thereby reducing the reliance on emergency care for initial cancer presentations. Future research should identify the factors associated with ED use prior to cancer diagnosis and assess interventions for improving early detection and timely outpatient care.

## Notes

Supplement 1. **eMethods.** Additional Information Regarding Data Sources and Covariates **eFigure 1.** Study Flow Diagram **eTable.** Baseline Characteristics of Unmatched Patients With and Without an Emergency Department Visit in the 90 Days Prior to Cancer Diagnosis **eFigure 2.** Hazard of Death for Patients Admitted Versus Discharged on the ED Visits Compared to Matched Patients Without an ED Visit Prior to Cancer Diagnosis

Supplement 2.
Data Sharing Statement
