# Supplementary material for: Emergency Department Use Prior to Cancer Diagnosis and Mortality
Source: JAMA Netw Open. 2025 Jul 22;8(7):e2522585. doi: 10.1001/jamanetworkopen.2025.22585 (PMC12284740; doi:10.1001/jamanetworkopen.2025.22585)
Supplement: Supplement 1. — eMethods. Additional Information Regarding Data Sources and Covariates eFigure 1. Study Flow Diagram eTable. Baseline Characteristics of Unmatched Patients With and Without an Emergency Department Visit in the 90 Days Prior to Cancer Diagnosis eFigure 2. Hazard of Death for Patients Admitted Versus Discharged on the ED Visits Compared to Matched Patients Without an ED Visit Prior to Cancer Diagnosis [file jamanetwopen-e2522585-s001.pdf]

Supplemental Online Content

Grewal K, Calzavara AJ, McLeod SL, et al. Emergency department use prior to cancer diagnosis and mortality. *JAMA Netw Open*. 2025;8(7):e2522585.
doi:10.1001/jamanetworkopen.2025.22585

eMethods. Additional Information Regarding Data Sources and Covariates

eFigure 1. Study Flow Diagram

eTable. Baseline Characteristics of Unmatched Patients With and Without an Emergency Department Visit in the 90 Days Prior to Cancer Diagnosis

eFigure 2. Hazard of Death for Patients Admitted Versus Discharged on the ED Visits Compared to Matched Patients Without an ED Visit Prior to Cancer Diagnosis

This supplemental material has been provided by the authors to give readers additional information about their work.

© 2025 Grewal K et al. *JAMA Network Open*

eMethods. Additional Information Regarding Data Sources and Covariates

Data Sources

To identify patients with a history of cancer, the Ontario Cancer Registry (OCR) was used. The OCR is a registry that contain all the diagnosed cases of cancer (expect squamous and basal cell carcinoma) in the province.[1] ED visits were identified from the Canadian Institutes of Health Information National Ambulatory Care Reporting System (CIHI-NACRS). NACRS is an administrative database that contains anonymized, abstracted data on all ED patient visits in the province of Ontario; it contains over 300 data points on every ED visit. Reporting to NACRS is mandatory in Ontario. Data in NACRS are reviewed and errors and/or missing data are identified, and returned to the submitting hospital as necessary for resubmission; therefore, missing data for mandatory variables in NACRS is very low.[2] CIHI's Discharge Abstract Database (DAD) captures information on all acute care hospitalizations and in-patient surgical procedures in the province. The Ontario Health Insurance Plan (OHIP) database contains all physician billings in the province. The Immigration, Refugees and Citizenship Canada (IRCC) database was used to identify immigration status. The Registered Persons Database contains validated demographic information and vital statistics, such as mortality for all Ontario residents, including out-of-hospital deaths.[3]

Covariates

Validated algorithms were used to identify patient comorbidities in the linked databases.[4-7] To examine rurality, the Rural Index of Ontario (RIO) score was used. The RIO score is a continuous score calculated by Statistics Canada, and is assigned to each patient based on postal code.[8] The score incorporates: population density, distance to basic referral centre, and distance to advanced referral centre. A higher RIO score is indicative of a more rural area, with scores greater than 40 considered to be rural based on funding models by the Ministry of Health and Long Term Care.

eReferences

1. de Oliveira C, Bremner KE, Pataky R, et al. Understanding the costs of cancer care before and after diagnosis for the 21 most common cancers in Ontario: A population-based descriptive study. CMAJ Open. 2013:**1**(1):E1-8.
2. Canadian Institute for Health Information. CIHI data quality study of Ontario emergency department visits for fiscal year 2004–2005: Executive summary. CIHI: Ottawa, ON. 2008.
3. Iron K, Zagorski BM, Sykora K, Manuel DG. Living and dying in Ontario: An opportunity for improved health information. http://www.ices.on.ca/file/Living_and_dying_in_Ontario_March19-08.pdf. 2009. Accessed Sept 26, 2024
4. Gershon AS, Wang C, Guan J, Vasilevska-Ristovska J, Cicutto L, To T. Identifying individuals with physician diagnosed COPD in health administrative databases. *COPD*. 2009;**6**(5):388-394.
5. Hux JE, Ivis F, Flintoft V, Bica A. Diabetes in Ontario: Determination of prevalence and incidence using a validated administrative data algorithm. *Diabetes Care*. 2002;**25**(3):512-516.
6. Schultz SE, Rothwell DM, Chen Z, Tu K. Identifying cases of congestive heart failure from administrative data: A validation study using primary care patient records. *Chronic Dis Inj Can*. 2013;**33**(3):160-166.
7. Tu K, Campbell NR, Chen ZL, Cauch-Dudek KJ, McAlister FA. Accuracy of administrative databases in identifying patients with hypertension. *Open Med*. 2007;**1**(1):e18-26.
8. Kralj B. Measuring rurality - RIO2008_BASIC: Methodology and results. OMA: Toronto, ON. 2009.

eFigure 1. Study flow diagram

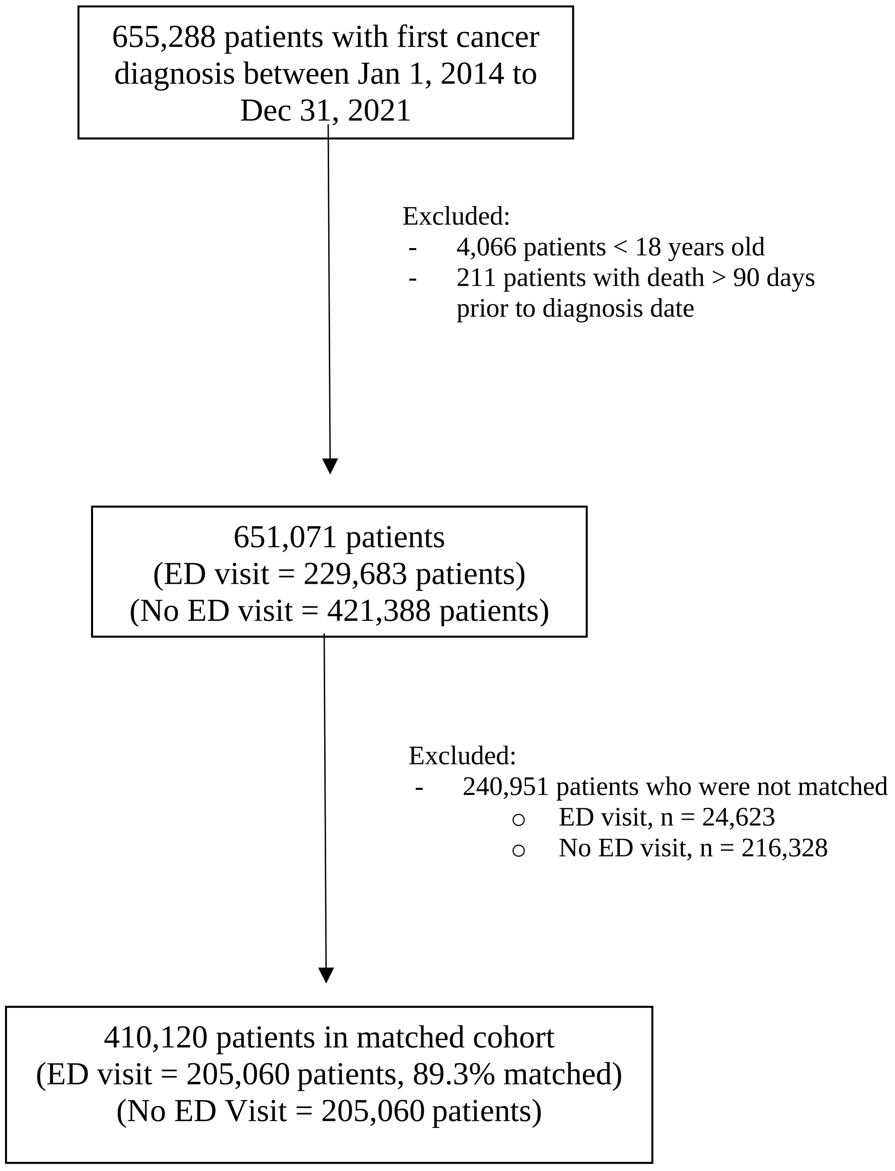

© 2025 Grewal K et al. *JAMA Network Open*

eTable. Baseline characteristics of unmatched patients with and without an emergency department visit in the 90 days prior to cancer diagnosis

Characteristic, n(%)		ED visit in the 90 days prior to cancer diagnosis	
		Yes (n=24,263)	No (n=216,328)
Age	18-55	1,544 (6.3%)	80,562 (37.2%)
	55-64	2,599 (10.6%)	53,897 (24.9%)
	65-74	4,454 (18.1%)	56,071 (25.9%)
	75+	16,026 (65.1%)	25,798 (11.9%)
Sex	Female	12,446 (50.5%)	143,518 (66.3%)
	Male	12,177 (49.5%)	72,810 (33.7%)
Rural	No	20,875 (84.8%)	201,216 (93.0%)
	Yes	3,700 (15.0%)	14,622 (6.8%)
	Missing	48 (0.2%)	490 (0.2%)
Material resources quintile	1 (highest resources)	3,238 (13.2%)	56,308 (26.0%)
	2	3,965 (16.1%)	49,506 (22.9%)
	3	4,525 (18.4%)	41,976 (19.4%)
	4	5,308 (21.6%)	36,467 (16.9%)
	5 (lowest resources)	7,222 (29.3%)	30,773 (14.2%)
	Missing	365 (1.5%)	1,298 (0.6%)
Household and dwellings quintile	1 (highest stability)	2,627 (10.7%)	46,217 (21.4%)
	2	3,374 (13.7%)	43,903 (20.3%)
	3	4,578 (18.6%)	40,801 (18.9%)
	4	5,603 (22.8%)	38,131 (17.6%)
	5 (lowest stability)	8,076 (32.8%)	45,978 (21.3%)
	Missing	365 (1.5%)	1,298 (0.6%)
Immigration, last 5 years	No	21,126 (85.8%)	178,610 (82.6%)
	Yes	3,378 (13.7%)	33,049 (15.3%)
	Unknown - n (%)	119 (0.5%)	4,669 (2.2%)
Ontario Health Region	West	7,623 (31.0%)	62,292 (28.8%)
	Central	4,481 (18.2%)	54,187 (25.0%)
	Toronto	4,669 (19.0%)	42,335 (19.6%)
	East	5,383 (21.9%)	46,152 (21.3%)
	North East	1,834 (7.4%)	8,254 (3.8%)
	North West	*628-632	2,991 (1.4%)
	Missing	*1-5	117 (0.1%)
Comorbidities	CAD	6,374 (25.9%)	13,190 (6.1%)
	CHF	8,848 (35.9%)	2,779 (1.3%)
	COPD	7,096 (28.8%)	5,993 (2.8%)
	Dementia	4,637 (18.8%)	1,726 (0.8%)
	Diabetes	10,176 (41.3%)	32,609 (15.1%)
	Hypertension	19,357 (78.6%)	86,041 (39.8%)
	Stroke	3,564 (14.5%)	1,751 (0.8%)
Any ED visit 6-30 months pre-index		17,627 (71.6%)	64,038 (29.6%)
Any hospitalization 6-		9,058 (36.8%)	19,374 (9.0%)

© 2025 Grewal K et al. *JAMA Network Open*

30 months pre-index			
Usual provider of care (UPC) type	No visits	624 (2.5%)	12,364 (5.7%)
	1-2 visits	998 (4.1%)	17,920 (8.3%)
	No UPC, no GP visits	169 (0.7%)	855 (0.4%)
	No UPC, saw GP	9,569 (38.9%)	72,868 (33.7%)
	UPC, specialist	1,504 (6.1%)	16,604 (7.7%)
	UPC, GP	90,453 (44.1%)	91,889 (44.8%)
Cancer type	Bladder/urinary	335 (1.4%)	6,440 (3.0%)
	Breast	*1-5	65,963 (30.5%)
	Colorectal/small intestinal	2,129 (8.6%)	4,373 (2.0%)
	Gastroesophageal	1,081 (4.4%)	*41-45
	Gynecological	55 (0.2%)	50,178 (23.2%)
	Head/Neck	*17-21	24,209 (11.2%)
	Hematologic/Lymphoma	3,315 (13.5%)	1,763 (0.8%)
	Liver/gall bladder	2,237 (9.1%)	0 (0.0%)
	Male genitourinary	32 (0.1%)	42,220 (19.5%)
	Neurological	4,475 (18.2%)	*1-5
	Other/Unknown	426 (1.7%)	18,838 (8.7%)
	Pancreatic	4,015 (16.3%)	0 (0.0%)
	Renal	223 (0.9%)	2,298 (1.1%)
	Thoracic	6,278 (25.5%)	0 (0.0%)
Diagnosis year	2014	3,139 (12.7%)	26,919 (12.4%)
	2015	3,356 (13.6%)	27,549 (12.7%)
	2016	2,792 (11.3%)	28,263 (13.1%)
	2017	3,205 (13.0%)	28,976 (13.4%)
	2018	2,969 (12.1%)	29,021 (13.4%)
	2019	2,864 (11.6%)	27,915 (12.9%)
	2020	3,848 (15.6%)	20,739 (9.6%)
	2021	2,450 (10.0%)	26,946 (12.5%)

*note small cells and other cells have been supressed to prevent back calculation.

© 2025 Grewal K et al. *JAMA Network Open*

eFigure 2. Hazard of death for patients admitted versus discharged on the ED visits compared to matched patients without an ED visit prior to cancer diagnosis

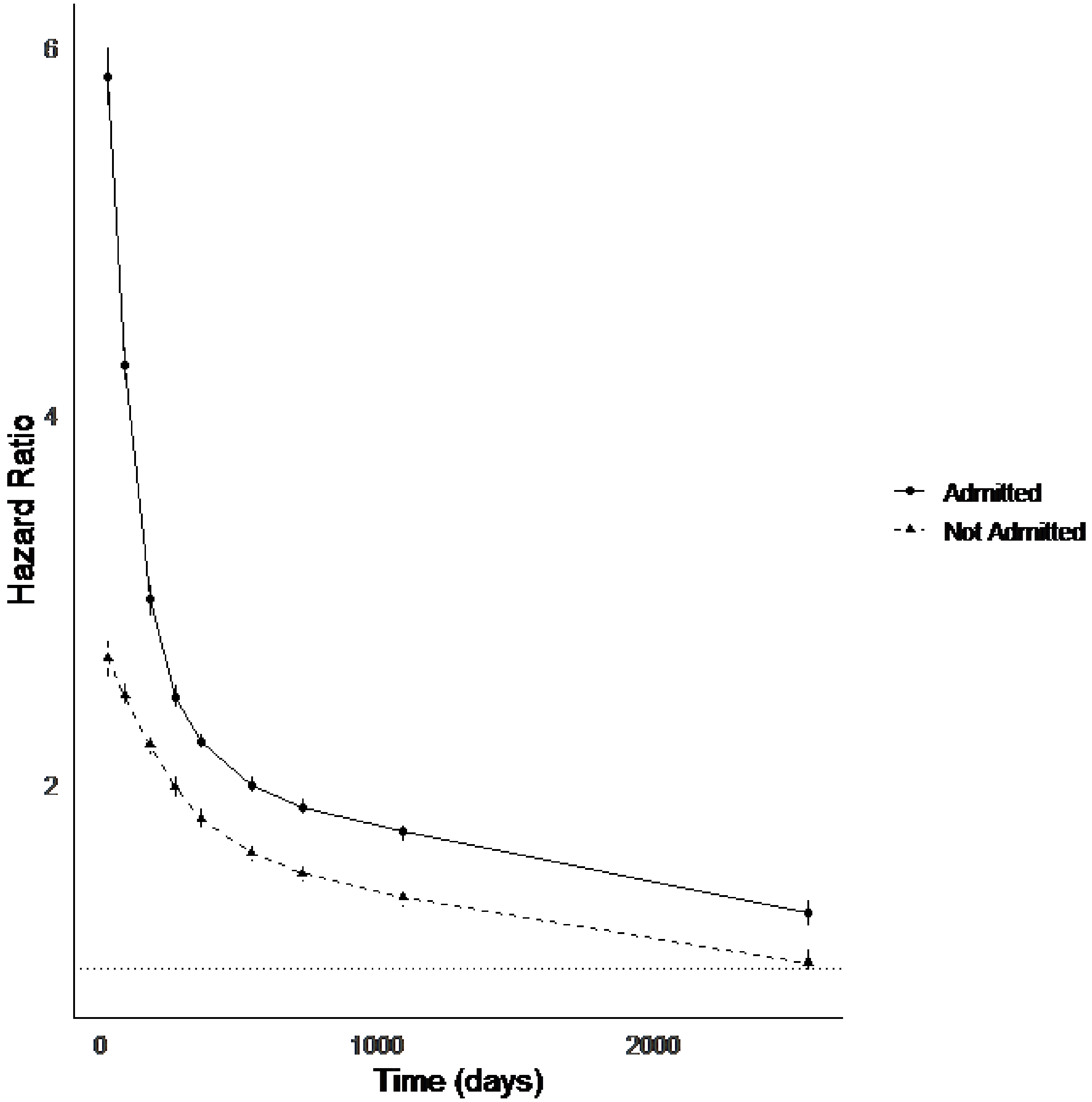

© 2025 Grewal K et al. *JAMA Network Open*
