# Supplementary material for: Emergency Department Use Prior to Cancer Diagnosis and Mortality
Source: JAMA Netw Open. 2025 Jul 22;8(7):e2522585. doi: 10.1001/jamanetworkopen.2025.22585 (PMC12284740; doi:10.1001/jamanetworkopen.2025.22585)
Supplement: Supplement 2. — Data Sharing Statement [file jamanetwopen-e2522585-s002.pdf]

Grewal. Emergency Department Use Prior to Cancer Diagnosis and Mortality. *JAMA Netw Open*. Published July 22, 2025. doi:10.1001/jamanetworkopen.2025.22585

Data Sharing Statement

Data available: No

Explanation for why data not available: The dataset from this study is held securely in coded form at ICES. While data sharing agreements prohibit ICES from making the dataset publicly available, access may be granted to those who meet pre-specified criteria for confidential access, available at www.ices.on.ca/DAS. The full dataset creation plan and underlying analytic code are available from the authors upon request, understanding that the computer programs may rely upon coding templates or macros that are unique to ICES and are therefore either inaccessible or may require modification.
